# Analysis of Determinants of Readiness for Professional Development Among Polish Nurses

**DOI:** 10.3390/ijerph16101800

**Published:** 2019-05-21

**Authors:** Anna Bartosiewicz, Edyta Łuszczki, Andrzej Różański, Małgorzata Nagórska

**Affiliations:** 1 Institute of Nursing and Health Sciences, Faculty of Medicine, University of Rzeszow, 35-959 Rzeszow, Poland; eluszczki@ur.edu.pl; 2 Department of Labour Pedagogy and Andragogy, Faculty of Pedagogy and Psychology, Maria Curie-Sklodowska University, 20-004 Lublin, Poland; rozanski@umcs.pl; 3 Institute of Experimental and Clinical Medicine, Medical Faculty, University of Rzeszow, 35-959 Rzeszów, Poland; nagorska@ur.edu.pl

**Keywords:** nursing staff, professional development, readiness, lifelong learning

## Abstract

The continuous development of medical sciences and the introduction of new diagnostic methods and treatment with the use of specialized equipment means that the knowledge and skills acquired during university studies are no longer sufficient. This obliges nursing staff to raise their professional qualifications in order to provide the appropriate quality of medical services. The aim of the study was an analysis of nurses readiness for learning and development and factors determining this readiness. The study was conducted among 756 nurses. The questionnaire method adopted was the readiness of employees for learning and development (RELD) standardized questionnaire, and a questionnaire containing sociodemographic data of the respondents. For the subscales of readiness for learning and development, average results were predominant and concerned in particular the level of openness to changes in environment (A1 scale), and self-evaluation of past educational development (C5 scale). The readiness of the nurses examined to learn and develop was on an average level for all the subscales. Younger nurses, with a lower seniority, having higher education and additional qualifications had a higher readiness for learning and development.

## 1. Introduction

**Figure 1:**
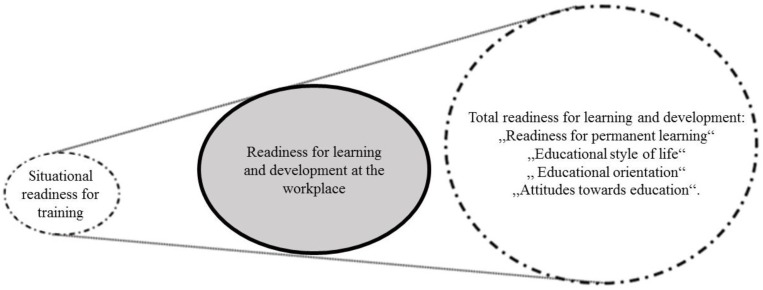
Theoretical categories of the concept of readiness for learning and development [3].

Lifelong learning is becoming an economic imperative, and acquiring new skills is crucial in many professions in this age of technological change, given its demography and the labor market [1,2]. Readiness is defined as the ability to undertake a certain activity and as an index it reflects the scale of actions undertaken by individuals in order to continue their education and career development, to complement and enrich professional practice [3,4]. This is accurately described by Herbert: “The capacity to learn is a gift; the ability to learn is a skill; the willingness to learn is a choice” [5]. According to the author of the questionnaire used in the present study, readiness is the consent to being available at the workplace, understood as giving the employer the disposal of the individual’s abilities and time [3]. He distinguishes three concepts referring to adults learning: Situational readiness (understood as a single educational event), readiness to learn and develop in the workplace (professional development), and readiness to develop in the aspect of lifelong learning (understood broadly—as a lifestyle) [3] (Figure 1).

Lifelong learning is a priority in European policy on adult education. In the context of dynamic technological change, globalization and demographic change, lifelong learning is considered even more important than ever before [6,7,8].

Permanent acquisition of knowledge and improvement of skills is a condition that enables effective functioning in a changing reality that puts the necessity of learning into every profession [9]. Dynamic development in medicine puts high demands on health care workers, and updating knowledge and postgraduate education is of particular importance [10,11]. This is also underlined in the 2013 UNESCO report, indicating the importance of lifelong learning in the field of health [12].

The continuous development of medical sciences as well as the introduction of new diagnostic methods and treatment with the use of specialized equipment make the knowledge and skills acquired during graduate studies insufficient [13,14]. Lifelong learning has also been recognized as a necessity for the nursing profession [15]. The "Future of Nursing" report indicates the need for an adequate level of education that would provide the required level of knowledge of nursing staff [16]. Over the last three decades, nursing in many countries around the world has become a more complex discipline that requires more skills. Undoubtedly, the educational policy of the European Union and the resulting Bologna Declaration as well as the Tunning Educational Structure in Europe Project contributed to the development of a uniform area of education in this field [17]. Nurses are increasingly perceived as an independent professional group with sufficient knowledge and skills in managing patient care [18]. This is especially important in the situation of a shortage of medical personnel, which affects many countries and attracts the attention of governments with regards to efficiency and rational resource management [19]. The changes taking place in the nursing profession are as follows: Taking over more and more responsible tasks, writing prescriptions and referrals for examination, as well as providing consultancy to patients [20,21]. One of the most important forms of professional development is self-education, which allows for a personal development that is conscious and tailored to individual needs [11]. However, an essential condition guaranteeing success in this field of science is the strong will and motivation of the learner [22]. In order to ensure adequate standards of care for patients and to strengthen their professional status, sustainable development based on readiness for learning and development is required. Graf in her own article indicates the direct impact of professional development of nurses not only on the quality of medical services provided, but also on the image of the entire nursing environment [23]. Fancenloben points to the important role of the compensatory function in the continuous education of nurses, which allows one to supplement knowledge at every stage of work and in constantly changing conditions, often related to the change of place of work and residence. This is particularly important in the processes of reorganizing the workplace and the widespread globalization of society [24]. Analyzing the current situation on the labour market, the structure of societies, demographic trends and staffing requirements in the health care systems of many countries, a thesis can be put forward that nursing throughout the world is gaining importance as a substitute for primary health care which is a prerequisite for learning and development among nurses [19]. The study presented in this article and the analysis of the scientific literature in this area corresponds with the WHO guidelines found in Global strategic directions for strengthening nursing and midwifery 2016–2020. This document sets out a global strategy of actions and a framework for strengthening nursing and midwifery services to help countries achieve universal health coverage and the Sustainable Development Goals [25]. 

Global trends in this field, oblige the nursing staff to upgrade their professional qualifications and thus to ensure the appropriate quality of medical services provided [24,26].

The present study investigated the readiness of Polish nurses to learn and develop. It offers answers to the questions about which factors determine the willingness of nursing staff to improve their skills. And can help managers of healthcare entities to better control and deal with human capital in the units of the health care sector. 

The aim of the study was as follows: Analysis of the readiness for learning and development among nurses in the South-Eastern part of Poland depending on selected sociodemographic factors that determine this readiness.

## 2. Materials and Methods 

### 2.1. Ethics

This research project was carried out in accordance with the Helsinki Declaration. The study was approved by the Bioethics Committee at the University (Resolution number 12/12/2015) and by all appropriate administrative bodies.

### 2.2. Study Group

The descriptive study was conducted among 756 Primary Health Care nurses in 2017, among randomly chosen treatment entities in the South-East part of Poland. A total of 756 nurses agreed to participate in the study. The examined respondents were a representative sample (25%) of all primary health care nurses (3023) employed in this region of Poland.

Inclusion criteria:

- Nurses currently employed.
- Nurses with a minimum of one year of professional experience.
- Nurses present and willing to participate in the study during data collection period.
- Completely filled in questionnaires.

Exclusion criteria:

- Nurses who were not qualified to practice.
- Nurses with less than the minimum of one years of work experience.
- Other employees of health system.
- Nurses who were unwilling to participate in the study.
- Incomplete questionnaires were not considered.

#### Characteristics of the Study Group

The average age of the respondents was 47.76 ± 9.65 years and ranged from 22 to 69 years. Almost half of the respondents—343 (45.4%) were nurses with secondary nursing education. The second group, in terms of numbers were nurses with the professional title of bachelor of nursing—higher vocational education—157 (20.8%). Finally, the third group; people with higher education—104 (13.8%). A nursing education and specialisation in nursing was shown by 73 (9.7%) people, 38 (5.0%) of the nurses held a 1st degree and specialization in nursing. Only 41 (5.4%) of participants had a second-level Master’s degree and a specialization in nursing. Over half of the respondents—411 (54.4%) had been working in the profession for over 20 years. More than every fourth person—159 (21.0%) had been employed from 16 to 20 years. Less numerous groups of nurses have been employed in the profession: For 1–5 years—47 (6.2%), from 6 to 10 years—68 (9.0%) and from 11 to 15 years—71 (9.4%). 

The most frequently mentioned health care facility in which nurses had basic employment were public health care institutions—full time contract—644 (85.2%). In non-public healthcare institutions, 112 (14.8%) of the respondents were contract workers. Just over half of nurses—404 (53.4%) had a specialist course, 345 (45.6%) had a qualification course in the field of nursing, almost every third respondent completed a training course—247 (32.7%). There were 141 (18.7%) people specializing in nursing, and 59 (7.8%) nurses had other forms of postgraduate improvement.

### 2.3. Research Technique and Tools

Two questionnaires were used for data collection. The first part was a demographic questionnaire including items about age, education, work experience, self-assessment of heath, form of employment, marital status, maternity experience, place of residence or place of work. The second part was a standardized questionnaire: Readiness of employees for learning and development (RELD, 2012). This questionnaire does not contain questions about sociodemographic data of respondents, only questions related to pro-development readiness of adults.

The selected method of research for this study was a diagnostic survey carried out using a questionnaire technique.

**Figure 2:**
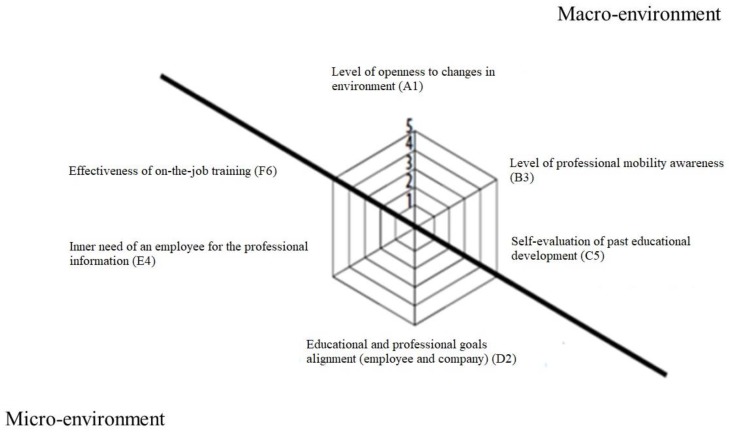
Dimensions of readiness for learning and professional development of employees [2].

The RELD questionnaire (version 2012-Publisher UMCS, Lublin, Poland.) was used to assess the employees’ pro-development readiness. The tool, thanks to the applied five-point Likert scale, allows measuring the readiness for learning and development in the workplace. The validity of the questionnaire was evaluated by three competent judges (specialists in the field of career guidance, andragogy and human resource management). The applied Likert scale enabled the measurement of the acceptance level of the content proposed in 48 statements. On the basis of the factor analysis carried out, out of 48 statements, six theoretical subscales (corresponding to the studied areas) were identified [3] (Figure 2).

The first three subscales were included in the group of so-called general predispositions for development and learning, in which the subscales were distinguished: The level of openness to changes taking place in the environment (12 statements), the level of professional mobility (4 statements), and self-assessment of the effectiveness of educational and professional goals (7 statements). The next three subscales focused on the aspects of the so-called "professional readiness for learning and development": A subscale of the felt community of educational and professional goals (9 statements), the level of professional information demand (3 statements), and the level of effectiveness of in-service training (4 statements). The remaining nine statements served as a buffer function. The subscales were described by following statements:

**A1—The level of openness to changes in environment**: This included the individual’s self-knowledge described by wording: I have many interests; I am interested in new ideas for solving emerging problems; it makes me happy to learn new things; I do not care about my comprehensive non-professional intellectual development; I have innate intuition, thanks to which I achieve a lot; I have enough potential to take on completely new challenges; regardless of the situation at work, I try to constantly improve my professional qualifications; I try to draw conclusions from any situation that would help in achieving my future goals; I actively look for opportunities to expand my knowledge and skills; I would like to have an impact on the subject of training organized in my company; I am happy to report my training needs to my superiors; it is good that people have to learn all their lives.

**B3—Level of professional mobility awareness:** This allowed individuals to comment on the perception of their professional opportunities in a macro-environment: A difficult situation on the labor market significantly limits my job opportunities; people of my age have little chance of satisfactory work; gender plays a significant role in developing professional careers; men have a better chance of finding an interesting job than women.

**C5—Self-evaluation of past educational development:** This allowed individuals to respond to their own educational experiences in the context of achieved results. They focused on the following statements:

I am well-educated; I have made full use of my own professional development opportunities; I am satisfied with my education; I do not assess my current educational / self-education activity very much; education is a significant value in my life; with learning in the past, I usually achieved very good results/assessments; being in the role of a student, I always apply to learning to achieve satisfying results.

**D2—Educational and professional goals alignment (employee and company):** This enabled assessment in the following areas: Whether the current job fully matches my potential; the employer is interested in my professional development; in connection with the work I am required to improve my qualifications; the employer supports me in my professional development concept; without the help of an employer, I would rather not invest myself in my own development; the training policy in the company suits me perfectly; in case of problems I know who to ask for help in the company; the employer is interested in my being educated not only in terms of work; the industry in which I work has a future.

**E4—Inner need of an employee for the professional information:** Statements included in this subscale were: Knowledge and skills that I have are perfectly adequate for my professional needs; in my situation, learning things that do not bring professional benefits do not make sense; I usually learn in situations when it is related to my current or future professional work.

**F6—Effectiveness of on-the-job training:** The points of the subscales contained the following statements: Training in the company is generally a waste of time; I feel compelled to participate in training I am not interested in; due to the workload I have too little time for pro-development activities; I get the impression that I take part in too many training sessions.

The Cronbach’s alpha reliability index for the entire scale was 0.674 [2,27,28].

In order to check the validity of the completed questionnaires in terms of understanding the questionnaire, a pilot study was conducted among 34 Primary Health Care nurses. The median age of the respondents was between 23–55 years (38.32 ± 9.81), 58.8% of them were married, 64.7% were nurses with a minimum of 1st degree studies and a specialization. The pilot study used both the author’s and RELD questionnaire. The reliability of the RELD questionnaire subsystem fluctuated within the Cronbach alpha range of 0.284 (E4 scale) to 0.926 (A1 scale). Similarly, it was found that the consistency of individual questions when building the scale was satisfactory, as most of the questions correlated positively with each other, and only in a small number of cases was the correlation weakly negative. Similarly, the correlations of particular subscale items with the general results were mostly strongly positive and confirm a good understanding of the tool.

The full research study was conducted among 756 Primary Health Nurses after obtaining approval from the managers of these facilities. The purpose of the study was explained at meetings. The respondents received oral information about the study and then written information about its purpose and its voluntary nature. The respondents were assured that their consent or refusal to participate would not affect their continued employment in a given health care institution. To ensure data confidentiality, the questionnaires were numbered and placed in sealed envelopes, they were then handed to the respondents directly during one-to-one meetings. Correctly completed questionnaires were acknowledged as consent of the participants to participate in the study. A total of 1320 questionnaires were distributed, of which 800, i.e., 60%, were collected back. After verifying all the questionnaires, 44 questionnaires were rejected due to the incompleteness of the answers provided. Finally, the data from the 756 questionnaires were plotted on the sheet and analyzed statistically. 

### 2.4. Statistical Analysis 

The estimation method and the following statistical methods were used:

1. In order to present statistical data, the method of descriptive statistics was used: The arithmetic mean (M), the value of which determines the average level of a given variable and the standard deviation (SD), the statistical measure of the results spreading around the expected value.
2. Differences between quantitative variables were tested using the Mann—Whitney test and Kruskal—Wallis test for calculating the Spearman rho correlation coefficient. This was related to the lack of normality of quantitative variable distributions (verified by the Kolmogorov—Smirnov and Shapiro—Wilk tests) and the lack of parallelity of the compared groups (verified by the test2 compliance test and the binominal test).

The level of significance was *p* < 0.05.

The calculations were carried out with the IBM SPSS Statistics 20 program (IBM, Armonk, NY, USA).

## 3. Results

As shown in Table 1, the nurses presented the highest level of readiness for learning and development of in the scope of openness to changes taking place in the environment (3.76 ± 0.49), where half of the respondents obtained no more than 3.75 points, on a scale of 1–5 points. To a slightly lesser extent (3.68 ± 0.54), the respondents of the level of self-evaluation of past educational development and of effectiveness of on-the-job training (3.60 ± 0.52). The average level of the educational and professional goals alignment (employee and company) was 3.33 ± 0.54 points. The nurse grouped lowest were in the case of the level of inner need of an employee for the professional information (2.95 ± 0.48) and the level of professional mobility awareness (2.88 ± 0.54).

**Figure 3:**
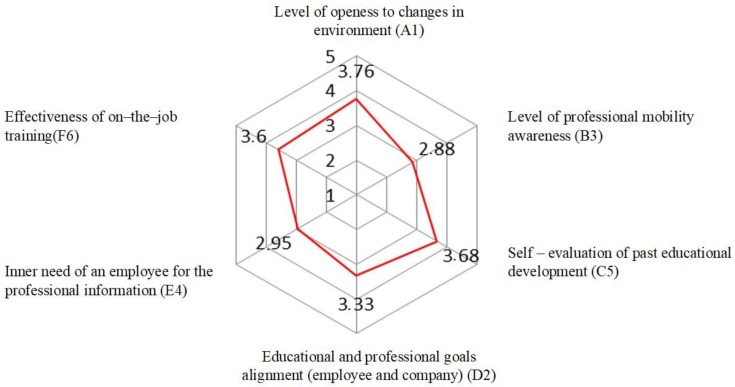
Dimensions of readiness for learning and professional development of employees—red line shows results.

For all subscales of readiness for learning and development, average scores prevailed (Figure 3). In particular, they concerned the following subscales:

- A1—level of openness to changes in environment—*N* = 602, i.e., 79.6% of all 756 surveyed nurses achieved these average results.
- C5—self-evaluation of past educational development—*N* = 591, i.e., 78.2% of all 756 surveyed nurses achieved these average results.
- F6—effectiveness of on-the-job training—*N* = 580, i.e., 76.7% of all 756 surveyed nurses achieved these average results.

Sociodemographic factors affecting readiness to learn and develop were as follows: Age, education, work experience, additional qualifications and the form of employment of the nurses surveyed. With age and seniority, readiness for learning and development decreased. (Table 2).

Significantly higher results were achieved by nurses with at least first-cycle education and specialization in nursing, and having additional qualifications. This also concerned subjects with very good or good health.

Persons working simultaneously on a contract of employment and self-employed workers presented a higher level of their own experienced professional mobility (B3) than the rest the respondents.

Factors such as marital status, maternity experience, place of residence or place of work did not significantly differentiate the respondents’ readiness for learning and development.

## 4. Discussion

According to Andrew Palmer, technical progress forces a stronger and continuous relationship between education and employment [1]. In order to meet the demands of a dynamically changing reality, it is necessary to improve one’s qualifications. The literature indicates that each additional year of study is associated with an 8—13% increase in the hourly remuneration rate [1,2]. Continuous and dynamic advances in medicine means the health sector needs to engage in personal and professional development [29,30,31].

The aim of the study was to analyze the readiness for learning and development among nursing staff and sociodemographic factors affecting this readiness.

The author’s own research showed that the highest level of readiness for learning and development was presented by nurses in the field of openness to changes taking place in the environment, in the perception of the effectiveness of educational and vocational goals and in the awareness of the effectiveness of in-service training.

The analysis of the results showed that for all levels of readiness for learning and development the average scores predominated and most frequently referred to the level of openness to changes in environment (A1), self-evaluation of past educational development (C5) and the level of effectiveness of on-the-job training (F6). Therefore, the readiness of nurses to learn and develop was related to the professional readiness of those surveyed. Similar results were obtained by the author of the RELD questionnaire used in the current survey among qualified employees [3].

A factor that significantly influenced the readiness of nurses to learn and develop was the age and seniority of the respondents. Older people had a lower level of experienced professional mobility (*p* < 0.0001), effectiveness of educational and vocational goals (*p* = 0.0002) and demand for vocational information (*p* < 0.0001), whereas people with work experience from 1 to 10 years had significantly higher scores on their professional mobility (*p* = 0.0007) than their long-time colleagues. The results obtained may be due to the fact that older people are less willing to update their professional knowledge and take up self-education than young ones. Similar results have been presented by other researchers who point to a natural decline in the ability to take up education among older people and a greater willingness of young people to develop in order to increase the possibility of getting a job or a better position [32]. The results seem to confirm the widespread belief that the interest in education is declining with the increase in seniority [33]. However, in the current situation, when demographic changes and an aging population also affect the nurses’ professional group, it will be extremely important to effectively motivate older employees to work and develop [34,35,36].

Significantly higher results in terms of general readiness for learning and development were achieves by nurses with at least undergraduate education and a specialization in nursing (A1 *p* < 0.0001; B3 *p* < 0.0001; C5 *p* < 0.0001), while people with additional competences showed an additional higher level of the feeling of community of educational and professional goals. It can therefore be assumed that taking up new rights will not be too heavy a burden for nurses with appropriate education. According to Andrew Palmer, the level of education is still a factor in determining the acquisition of a job and the unemployment rate decreases with the level of education [1,2]. In the United States, the unemployment rate decreases with increases in the level of education. In some countries, e.g., in Singapore, employees are offered scientific loans that they can use to improve their qualifications [1].

In addition, better educated people are characterized by a greater ability to adapt and react to changes in the environment and greater activity in training and courses. The data from the Postgraduate Education Center confirms the above as they show that the vast majority of participants (96%) had higher education [37].

Nurses with very good or good health obtained higher scores on general readiness for learning and development (A1 *p* = 0.0190; B3 *p* < 0.0001; C5 *p* = 0.0003); and in terms of the feeling of community of educational and vocational goals (D2 *p* = 0.0006) than nurses with average or unsatisfactory health conditions. Nurses working simultaneously on a contract of employment and self-employed workers presented a higher level of their own professional mobility (B3 *p* = 0.0003) than the other nurses researched in this group, while nurses working for many clients had significantly higher results in terms of general readiness for learning and development (A1 *p* = 0.0079, B3 *p* = 0.0197, C5 *p* = 0.0485). The obtained data may be associated with a sense of greater stability and financial security. The results of the research show that a bad financial situation is a significant obstacle to undertaking post-graduate education [37,38,39,40]. The motivation to acquire new skills as well as the ease of adaptation to the dynamically changing reality are issues affecting various professional groups, including those related to the health sector [41]. They are important both in the personal context of the employed, directly related to their professional development, and also through individual units, to the functioning of the entire sector. Due to the fact that the health care sector is a dynamic environment and subject to various changes, processes related to raising qualifications and the willingness of the staff employed to develop should be subject to further scientific exploration [10,26,42].

### Limitations of the Study

There are also potential limitations of the study that need to be taken into account when interpreting the results:

- The primary study limitation was the relatively high age of the respondents (the vast majority of Polish nurses are at this age).
- The research was carried out only in one Polish Subcarpathian province. It is worth considering the study expansion to other regions of the country.
- Most of the respondents had only a secondary medical education.
- A large number of questionnaires were not collected back.

## 5. Conclusions

In all the subscales, the readiness for learning and development of the nurses surveyed was at an average level and concerned mainly the level of openness to changes (A1), the level of self-evaluation of past educational development (C5), and the level of effectiveness of on-the-job training (F6). The age and seniority of the nurses surveyed significantly affected the readiness to learn and develop. These factors were of a negative nature. Nurses with higher education and additional qualifications had higher readiness to learn and develop oneself. 

There is a growing interest in improving qualifications around the world, and people seem to accept more and more the need to constantly re-start their training [43]. The degree of education at the beginning of a career is not enough for the entire professional life but requires constant development and updating [1].

The results obtained can be used to plan training and courses as well as to create special pro-development programs, which may increase the nurses’ involvement in personal and professional development.

## Figures and Tables

**Table 1:** Level of readiness for learning and development (RELD) of nurses surveyed.

RELD	Range	A1	B3	C5	D2	E4	F6
M		3.76	2.88	3.68	3.33	2.95	3.60
Mediana		3.75	3.00	3.71	3.33	3.00	3.50
SD		0.49	0.81	0.54	0.54	0.48	0.52
Minimum		2.17	1.00	1.86	1.67	1.33	1.25
Maksimum		5.00	5.00	5.00	5.00	4.67	5.00
Percentyls	25	3.50	2.50	3.29	3.00	2.67	3.25
	50	3.75	3.00	3.71	3.33	3.00	3.50
	75	4.08	3.50	4.00	3.67	3.33	4.00

A1—level of openness to changes in environment, B3—level of professional mobility awareness, C5—self-evaluation of past educational development, D2—educational and professional goals alignment (employee and company), E4—inner need of an employee for the professional information, F6—effectiveness of on-the-job training.

**Table 2:** Readiness for learning and development (RELD) and selected sociodemographic factors among 756 nurses.

Type of Scale	Age	Education	Work Experience	Additional Qualifications	Self-Assessment of Health	Form of Employment
A1	0.1863	<0.0001	0.7561	<0.0001	0.0190	0.0317
B3	<0.0001	0.0001	0.0007	0.0003	<0.0001	0.0003
C5	0.0002	<0.0001	0.0483	<0.0001	0.0003	0.0214
D2	0.8001	0.5871	0.1941	0.6100	0.0006	0.3315
E4	<0.0001	0.0363	0.0676	0.0482	0.4336	0.1398
F6	0.9188	0.1174	0.5221	0.6100	0.4422	0.1382

A1—level of openness to changes in environment, B3—level of professional mobility awareness, C5—self-evaluation of past educational development, D2—educational and professional goals alignment (employee and company), E4—inner need of an employee for the professional information, F6—effectiveness of on-the-job training.

## Data Availability

The data sets generated and analyzed during the current study are available in the University of Rzeszow Repository (http://repozytorium.ur.edu.pl) as an Excel file under the name of the first and also the other authors, and the title of our manuscript.

## Abbreviations

RELD	Readiness of Employees for Learning and Development
CPD	Continuum Professional Development
PHC	Primary Health Care
M	the arithmetic mean
SD	the statistical measure of the results spreading around the expected values.
p	The level of significance was *p* < 0.005
